# Understanding why health professionals are leaving the UK national health service (NHS) – A systematic review and narrative synthesis

**DOI:** 10.1177/13558196251384845

**Published:** 2025-10-08

**Authors:** Chukwunwuba R. Onyejesi, Tiffeny James, Kalpa Kharicha

**Affiliations:** 1 Department of Population Health Sciences, 4616 King’s College London, London, UK; 2 Institute for Lifecourse Development, 4918 University of Greenwich, London, UK; 3 NIHR Policy Research Unit in Health and Social Care Workforce, 4616 King’s College London, London, UK

**Keywords:** NHS, intention to leave, retention, workforce

## Abstract

**Background:**

There is a global health care workforce crisis with staff shortages and difficulties with recruitment and retention, including in the UK’s National Health Service (NHS). To address this, it is important to understand why people decide to leave the NHS. Previous reviews have focused on specific NHS professions and have rarely considered factors in other settings which attract staff away from the NHS. This review aimed to include all professions in a systematic review of factors which “push” clinical staff to leave, or consider leaving, the NHS and which “pull” them to other destinations.

**Methods:**

We searched PubMed, Web of Science, CINAHL, and EMBASE for peer-reviewed articles and Google Scholar for grey literature using search terms related to all NHS professions and intentions to leave the NHS. We included qualitative, quantitative, and mixed methods studies and analysed data using a textual narrative synthesis with an integrated design.

**Results:**

Thirty-two papers were eligible for inclusion. We identified four key push factors: (1) high job demands due to, for example, staff shortages and increased workload; (2) failing organisational structures including poor pay and limited opportunities for career development; (3) personal and emotional factors such as work-related health issues and poor work/life balance, and (4) wider factors, including Brexit. The majority of factors identified as being responsible for high turnover were related to job demands and the organisational structure within the NHS. Factors pulling people to other destinations were discussed less frequently than push factors, but included perceptions of better: pay, working conditions, and work/life balance in other countries. Limitations to the studies included in the review were that evidence on all NHS professions was not available, and many of the studies were based on data collected retrospectively with the risk of recall bias.

**Conclusion:**

Pull and push factors affect multiple NHS professions. Further comparative studies comparing the UK with other countries can help inform potential interventions to improve staff retention.

## Introduction

There is currently a critical shortage of healthcare workers across the world.^1^ Factors that have contributed to this include a rising demand for health care, an increase in age-related chronic illnesses^1^ and market liberation which allows healthcare workers to migrate from their home countries to work in foreign healthcare systems.^2^ Increasing vacancy rates affect the quality of care and can lead to longer waiting times for patients. Increased workload and pressures related to high vacancies also negatively affect staff by exposing them to more stressful working conditions, making them more prone to making mistakes.^3,4^ Maintaining a sufficient labour force to manage health care demands is a challenge faced by many countries,^5^ including the UK.^6,7^

In the UK, the publicly funded National Health Service (NHS) aims to provide universal coverage where access to health care is based on clinical need rather than ability to pay. However, staff shortages across all NHS professions are affecting the delivery of essential services.^8^ As of December 2024, the NHS in England reported approximately 106,432 vacancies, representing a vacancy rate of 7.2% across all staff groups. This marks a slight decrease from the 7.7% vacancy rate recorded in December 2023. The COVID-19 pandemic amplified the effects of staff shortages: stress, workload, time pressures and adverse physical and mental health outcomes.^9^ While staff retention rates have marginally improved, dropping by 2.4% among hospital and community healthcare workers between September 2022 and September 2024,^10^ insufficient staffing has increased NHS spending on temporary staff, which is both a costlier alternative to permanent staff and can reduce continuity of care. In 2019/20, £2.38 billion was spent on temporary staff.^8^ The cost of temporary staff to the NHS was reported as having increased to more than £10 billion in 2022/23.^11^ Whilst the number of full time NHS staff increased by 263,000 between 2010 and 2023, this has been offset by an increase in health care demand due to population ageing, multimorbidity, and increasingly complex care needs.^12^

To address staff shortages, the NHS has historically depended on the recruitment of both clinical and non-clinical professionals from lower- and middle-income nations, many of whom leave their country of origin in search of more favourable career opportunities.^13,14^ The rates of overseas recruitment have fluctuated over the years due to a range of reasons including UK immigration policy, NHS funding, and staff shortages.^15^ As of June 2023, about 1 in 5 NHS staff had a non-British nationality. The majority of foreign nationals come from Asia, Africa or the European Union.^16^ The UK NHS adheres to a code of practice that ensures that overseas recruitment is ethically responsible.^17^ However, there are plans to reduce reliance on foreign staff through training and recruitment efforts within the UK, as overseas recruitment has been identified as potentially contributing to mass migration^13,14^ as well as widening health inequalities between higher and lower income countries.^14,18^ Plans to address staff shortages in the NHS include increasing medical school training places to 15,000 a year, GP training places by 50%, adult nursing training places by 92%, training for clinical staff through apprenticeship routes by 15%, and dental training places by 40% all by 2031/32.^12^ Recruitment strategies alone are not sufficient to balance staff shortages^19^; problems of retention have been well-documented, with significant numbers of NHS staff reporting their intention to leave or actually leaving the NHS.^20–22^ For doctors that leave the NHS to practice abroad, the most popular destinations are Australia and New Zealand,^23^ and the majority of the over 12,000 UK registered nurses who opted to leave the UK NHS in 2022/2023 applied to register in Australia, New Zealand and the United States.^24^

A 2023 report on why NHS staff leave collected data from 17,686 survey responses and 65 interviews over 19 months.^25^ The three factors rated as most important in terms of their influence on staff leaving the NHS were: stress (66%), staff shortages/resources (62%), and pay (55%).^25^ Other factors included: the organisational impact on staff members’ ability to provide good patient care, having unsupportive managers, and the impact staff felt of their work on their mental health.^25^ High turnover has a negative impact on the morale of staff that stay in post, and projects a negative image of the organisation.^26^ It is also a major problem for organisations as it incurs costs of recruiting, inducting, and training new staff.^26^

A series of strikes by several NHS staff groups commenced in 2022 and persisted over a 2-year period due to claims of inadequate pay in the context of a rising cost of living; concerns over patient safety; and staff shortages.^27^ These strikes further exacerbated workforce challenges in the NHS.^28^ The financial cost of the strikes to the end of January 2024 was estimated at £1.5 billion due to additional spending on temporary staff to provide cover for critical services and the cost of cancelling and rescheduling appointments.^28^ The strikes affected patient waiting times with more than 1.3 million health care appointments being rescheduled.^28^ The dispute over pay raised concerns that staff would be both reluctant to join the NHS and to stay in post without improved terms and conditions.^27^

To address the workforce deficit, the NHS Long Term Workforce Plan has instituted a three-pronged approach: *Train*, *Retain,* and *Reform,* which aims to retain the 130,000 staff estimated as intending to leave the NHS over the next 15 years.^12^ To inform the implementation of this workforce plan, it is important to have an understanding of the factors which influence the intentions and decisions of people to leave the NHS. Previous reviews of such factors have mostly focused on doctors and nurses; either as broad groups^1,29^ or as specific specialties such as primary care doctors,^30,31^ mental health nurses,^32^ or hospital nurses.^33^ Whilst one review has included the broader NHS workforce, it focused on the relationship between labour force satisfaction, wages, and retention within the NHS, excluding studies exploring other factors affecting NHS retention, and did not clearly specify which staff groups were included.^34^ Other reviews have focused on the factors within healthcare professionals’ current situations that either “push” them to leave (typically perceived as negative factors) or “pull” them to stay (typically positive factors); but have rarely considered desirable factors in other settings, countries, or situations which “pull” people to those destinations. By identifying what NHS staff perceive as the benefits of leaving the NHS, insights can be gained as to what they value, and what could potentially be implemented within the NHS, to improve their retention. The aim of this systematic review is therefore to identify and synthesise the “push” and “pull” factors (as defined below) influencing healthcare staff of any speciality leaving or intending to leave the NHS between 2013 and 2023, and explore any differences in these factors between professions.

## Methods

We followed Preferred Reporting Items for Systematic Reviews and Meta-analysis (PRISMA) guidance^35^ for this systematic review. See Online Supplement 1 (Table S1) for the PRISMA checklist. No protocol was registered for this review.

### Eligibility criteria

We included peer reviewed articles and grey literature reporting findings from qualitative, quantitative, and mixed methods studies about the push and pull factors for current or previous healthcare staff from any profession or speciality leaving or intending to leave the NHS. In this review we define pull factors as anything in another situation, setting, or country which “pull” people from the NHS to that setting. There was no limit on the time period since leaving the NHS. To be included, studies had to be:

• based in the United Kingdom including England, Scotland, Wales and Northern Ireland
• reported in English Language
• published since 2013

Studies were excluded if they:

• included NHS and private health care provider staff, but did not distinguish between the two groups when reporting push and pull factors
• focused only on students’ reasons for not joining the NHS
• included participants who left one NHS role to take up another NHS role

### Search strategy

We searched electronic databases from 2013 to 2 December 2023 for Cumulative Index to Nursing and Allied Health Literature (CINAHL) and 3 December 2023 for EMBASE, PubMed, and Web of Science. We combined search terms relating to the NHS (i.e., National Health Service, NHS); leaving the NHS (e.g., resign, relocate, intention to leave etc.); and NHS staff role. For the latter, we searched for general staff terms and specific healthcare professions. See Online Supplement 1 (Table S2) for the search strategies. We searched Google Scholar for the words “NHS staff leaving PDF” on 17 December 2023 to identify grey literature. We included “PDF” as we found in our preliminary searches that relevant reports were typically PDF format and that without the inclusion of this term, grey-literature searches produced a large number of irrelevant results. We screened Google results until they were no longer relevant. We also checked the reference lists of peer-reviewed papers to identify further relevant papers.

### Study selection

We used Zotero referencing management software. Following removal of duplicates, the lead author screened all titles against the eligibility criteria, removing any studies that were ineligible. The lead author screened the abstracts of all remaining papers, excluding ineligible papers, discussing any uncertainties with another author. Finally, the full text articles of remaining articles were assessed against the eligibility criteria. Two authors independently screened a sample of 20% (n = 9) of full texts to standardise the screening process. We agreed about eligibility for 8 out of 9 (89%) papers and resolved our only disagreement through discussion. The lead author then screened the remaining full texts independently, discussing any uncertainties with another author.

### Data extraction and synthesis

For each study, we extracted the following information: author(s), publication year, methodology (qualitative, quantitative, or mixed methods), research methods, sample size, profession of NHS staff, and push and/or pull factors described. Two authors independently extracted data for a sample of 20% (n = 6) of the included studies, to standardise the extraction process. The lead author then extracted data from all remaining papers. We synthesised the findings using a textual narrative approach with an integrated design,^36^ transforming quantitative data into qualitative data and describing the push and pull factors narratively.^37^ References for all included articles are listed below in Table 1.

**Table 1.** Details of included studies.

Author and year	Study type and method	Sample number and profession	Push factor	Pull factors
Milner et al., 2021a^39^	Qualitative analysis of free text questionnaire comments	52 doctors	• No longer feeling welcome in the UK • Feelings of rejection • Brexit perceived as racism	
Milner et al., 2021b^40^	Quantitative survey	59 doctors	• Brexit	
Theodosius et al., 2020^41^	Quantitative cross-sectional study	118 nurses	• High levels of patient-focused emotional labour • High levels of depersonalization and surface acting	
Crossland et al., 2021^42^	Quantitative survey	19 NHS paediatric surgeons	• High caseload • Financial reasons • High levels of stress in NHS • Lack of research funding opportunities • Uncertain future of service through multiple national review processes • Brexit	The following were perceived as better abroad: • Pay • Work/life balance • Recognition • Research facilities & opportunities • Control over how service is delivered
Ryan et al., 2019^3^	Mixed methods-online survey/thematic analysis	1918 counsellors	• Contracts not being renewed • Redundancy • Undervalued profession • Lack of paid roles • Limited opportunities for career development or promotion • Experiences of bullying • Feeling ethically and morally compromised in their role • Unsupportive management • Unmanageable demands	• Finding alternate employment
Mills et al., 2023^43^	Mixed methods-questionnaire and thematic analysis	106 dental practices	• Inability to offer competitive salaries • High workload • Poor access to training • Poor transport links • Rise in cost of accommodation and cost of living	
Weyman et al., 2023^25^	Quantitative survey-pairwise comparison	1958 NHS health professionals	• Highest ranked factors • High work-related stress • Workload intensity • Staffing levels	
Wilson et al., 2021^44^	Mixed method study-online survey and semi structured interviews	210 junior doctors	• Lack of professional development • Expectations not matching realities • Burnout • Poor work/life balance • Bleak outlook working for the NHS • Lack of interest in retaining those who want to leave • Pay not matching level of responsibility	• Being valued overseas • Less administrative work abroad • Better opportunities abroad
Pathmanathan & Snelling, 2023^45^	Qualitative study: Semi structured interviews	17 doctors	• Increased administrative tasks • Poor work/life balance • Lack of autonomy • Unable to work in preferred location • Lack of support from supervisors • Bullying • Perceived institutionalised racism within the NHS	• Attraction towards alternative careers
Hood & Patton, 2022^46^	Quantitative cross-sectional survey	102 healthcare assistants	• Poor autonomy • Low relatedness (i.e. connectedness, belonging, supportive collegiate relationships)	
Smith et al., 2017^47^	Quantitative survey	3695 medical graduates; cohorts from 1974 to 1977	• High work-related pressures • Reduced job satisfaction	• Increased time for leisure or other interests • Retirement of a partner
Smith et al., 2018^48^	Qualitative semi-structured interviews	17 doctors	• Poor working environment • Low staff morale • Lack of mentorship • Bullying from senior doctors and uncertainty over how to raise concerns	• Ease of obtaining a visa abroad • Being more highly valued • Better working environment abroad
Owen et al., 2019^49^	Mixed method study: Questionnaire and qualitative analysis of free text	929 GPs	• High workload intensity • Lack of patient contact • Potential introduction of a 7-day working week and associated reduced job satisfaction • Increasing demands and complexity of patients • Increase in administrative tasks	
Cheng et al., 2020^51^	Quantitative survey	36850 healthcare practitioners	• Experiencing aggression from either colleagues or patients, but especially from colleagues	
Zhou et al., 2022^51^	Qualitative survey	348 GPs	• Diagnostic uncertainty (subjective perception of inability to provide accurate explanation for patients’ health problems) • Reporting attending work whilst sick more than once in a 12-month period	
Robson & Robson, 2024^52^	Quantitative survey	433 nurses	• Leader-manager exchange and low perceived organisational support	
Prosser & Achour, 2023^53^	Mixed method study	123 orthodontists (36 NHS; 82 private)	• Poor treatment from employer	
Fasbender et al., 2019^54^	Quantitative survey	361 nurses	• Poor on-the-job embeddedness • Low job satisfaction • High work-related stress	
Napier & Clinch, 2019^55^	Qualitative study: Narrative interviewing method	12 GPs	• Declining levels of professional autonomy • Increasing demands for healthcare • Increase in administrative tasks and time pressures	
Nursing & Midwifery council, 2020^56^	Quantitative survey	5639 nurses and midwives	• Retirement • Change in personal circumstances including declining physical and mental health • High work-related pressures • Bullying and perceived racism • Unsupportive management • High workload • Concern over quality of care given to patients • Inability to meet requirements for revalidation	• Caring commitments • Need for relocation
Doran et al., 2016^57^	Mixed method study- survey and qualitative interviews	143 GPs	• Organisational changes • Increased volume of work • Increased complexity of work • Too high administrative/non-clinical workload • Impacting patient-doctor relationship • Negative media portrayal • Lack of support e.g. conflicts with workplaces around funding, career progression, flexible hours, workload distribution • Bullying culture • Low job satisfaction • Negative impact on well-being	
Khan et al., 2019^58^	Cross sectional observational study	593 NHS consultants	• Poor job autonomy	
Dale et al., 2015^59^	Mixed methods: Quantitative cross-sectional study and thematic framework approach	1192 GPs	• Volume and intensity of workload • Time spent on unimportant tasks • Introduction of 7-day working week • Reduced job satisfaction • Increase in administrative and bureaucratic tasks • Extra responsibilities and tasks • Increased patient complexity and chronic ill health • Emotional impact Significant predictors of intention to leave*:* • Older age • Male gender (for those under age 55) • 10+ years of service • Not having one or more accountable role in addition to their main practice responsibilities	• Travel/work abroad • Study or research
Nightingale et al., 2021^19^	Semi-structured telephone interviews	44 radiographers	• Increased workload • Lack of flexibility in working terms and conditions • Pay not being commensurate with level of responsibility • Poor work/life balance • Limited career progression opportunities • Feeling undervalued	
Ocean & Meyer, 2023^60^	Quantitative longitudinal study	NHS workforce (number unclear)	• Poor work/life balance	
Spooner et al., 2017^61^	Mixed method study: Online survey and qualitative interviews	816 junior doctors	• Dissatisfaction with proposed contract	• Family connections abroad • Better work/life balance abroad
Payne et al., 2023^62^	Online survey	502 consultant urologists	• Poor health • Limitation to in lifetime allowance • High workload and workplace related stressors • Lack of flexible working conditions • Negative interactions with hospital management	
General Medical Council, 2021^63^		13,158 doctors	• Dissatisfaction with role/place of work/NHS culture • Burnout/work related stress	• Desire to move abroad
Sansom et al., 2016^64^	Qualitative study: Semi-structured interviews	23 GPs	• Increased workload • Dissatisfaction with organisational change • Increased administrative tasks • Ill health was a cause of early retirement • Negative media portrayal • Feeling undervalued	• Early retirement as a viable option for GPs • Wanting to retire while still being young • Dedication of time and energy to other vocational roles • Wanting to spend time with family
Gauld & Horsburgh, 2015^5^	Quantitative survey	632 doctors	N/A	• Better working conditions abroad • Better career opportunities abroad • Better quality of life abroad
Lambert et al., 2018^65^	Questionnaire survey	5291 doctors	• Uncertainty over proposed new contracts • Unfavourable political factors in the NHS • Training in the UK perceived as inadequate/limited • Low pay not commensurate with responsibility • Excessive working hours • Feeling undervalued • Low morale • Poor work/life balance • Lack of support from seniors/management • Concern over patient safety	• Desire to work abroad • Better lifestyle abroad
Leversidge, 2016^66^	Questionnaire survey	2719 midwives	• Unhappiness with staffing levels • Dissatisfaction with the quality of care they were able to give patients • High workload • Lack of support from managers • Dissatisfaction with working conditions	

### Quality assessment

We used the mixed method appraisal tool (MMAT) to assess and describe the quality of included studies.^38^ Two authors independently assessed the quality of a sample of 20% (n = 6) included studies to standardise the process. We agreed on 34/40 (85%) criteria, resolving the disagreements through discussion. The lead author then assessed the quality of all remaining papers. No studies were excluded based on their quality.

## Results

### Study selection

**Figure 1.**
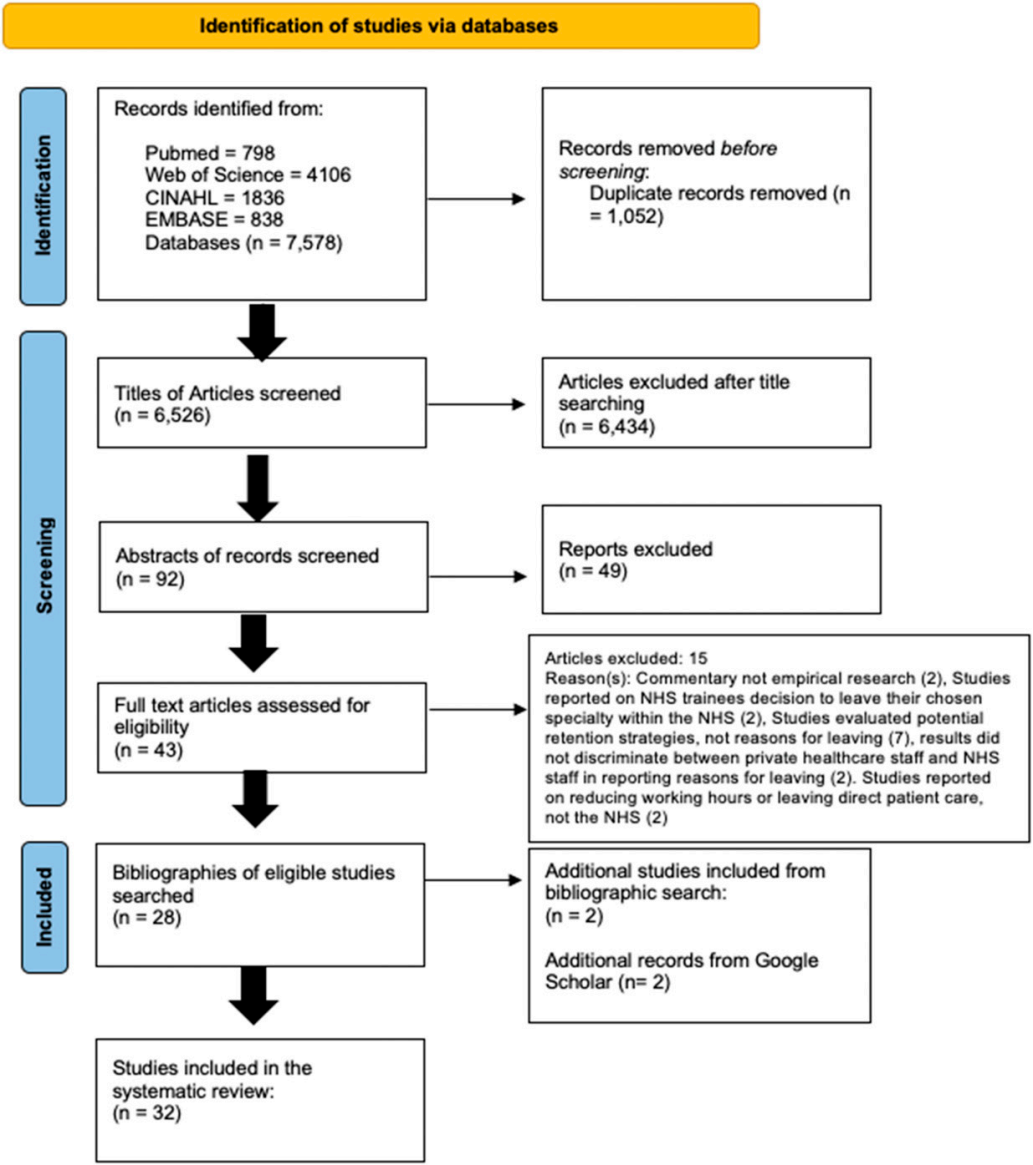
PRISMA flow diagram showing study selection process.

Searches on scientific databases produced 6526 results after duplicates were removed. We excluded 6434 based on their title, and a further 49 after reading the abstract. Out of the remaining 43 full texts, 28 were deemed eligible for inclusion. The main reason for excluding at this point was that articles explored potential retention strategies rather than reasons for leaving. We identified a further four eligible documents from the reference lists of included paper (n = 2) and grey literature searches (n = 2) bringing the total number of included papers to 32. See the PRISMA flow diagram for the study selection process (Figure 1).

### Quality of included studies

The qualitative and mixed method studies included in this review were generally of good quality. All qualitative studies included a clear statement of the research aims and applied appropriate qualitative research methods, namely semi-structured interviews. All mixed method studies integrated both qualitative and quantitative methods adequately. All studies included a rationale for using mixed method designs, which was to provide an in-depth exploration as to why NHS staff leave their employment. Assessment of the quality of mixed methods and qualitative studies are displayed in Online Supplement 1 in Tables S3 and S4 respectively. Around half of the studies included in this review (n = 17) were quantitative by design, including descriptive quantitative studies (Online Supplement 1; Table S5) and non-randomised studies (Online Supplement 1; Table S6) and were of varying quality.

### Summary of included studies

Of the 32 included studies, 17 were quantitative in nature, 7 were qualitative, and 8 used mixed methods. Data were collected from NHS staff who were either currently in post but were intending to leave, or had already left and were asked retrospectively to identify factors responsible for their decision. Nineteen papers commented on factors that determine why doctors working in the NHS either leave their employment or intend to leave. This included 7 focusing on General Practitioners (GPs), 2 on Junior Doctors, and 2 on NHS Consultants. Four studies involved Nurses across specialisms, and a further 2 focused on Midwives specifically. Three studies incorporated all NHS staff and the remaining studies focused on Dentists (n = 2), Counsellors (n = 1), Healthcare Assistants (n = 1), and Radiographers (n = 1). See table 1 for study details including the push and pull factors identified.

While the majority (n = 19) of papers included in this review explored reasons why NHS staff might opt to leave, 13 evaluated the potential effect of a pre-named exposure/factor on staff intentions to leave, including job satisfaction,^53^ job embeddedness,^54^ psychological morbidity,^58^ surface acting and emotional labour,^41^ aggression from both patients and staff,^50^ psychological needs fulfilment,^46^ national policy-led interventions,^49^ workplace stress,^3,55^ and the experience of Brexit.^39,40^ One study^25^ asked staff to rank the reasons for leaving from highest to lowest, 12 months following the experience of NHS working conditions post COVID-19.

### Findings

#### Push factors

The push factors reported in the studies included in this review were categorised into: high job demands; failing organisational structure; wider factors; and personal and emotional factors.

##### High job demands

Health professionals, irrespective of profession, leave the NHS due to high job demands, particularly due to high workload.^19,25,42,43,49,56,57,59,62,66^ High workload was exacerbated by staff shortages.^56,66^ Among GPs, increased workload was frequently cited as a direct consequence of an increase in administrative or non-clinical tasks due to complex referral pathways,^57,59,64^ as well as increasing demand for health care due to increased complexity of patients and chronic ill health.^49,55,59^ When compared to other recognised push factors on a scale to determine their relative importance, intensity of workload, work-related stress, and staffing levels ranked highest.^25^ Having such a high workload also raised concerns by health workers over the quality of care they are able to give patients^3,45,56,57,65,66^ usually as a result of increasing non-clinical workload^3,45,57,64^ or staff shortages.^56^

High workload was closely related to high work-related pressures and stress^3,42,47,54,62^ and understood to cause ill health such as anxiety and depression^56,57^ as well as burnout.^3,44,55^ High workload was also identified as increasing the risk of mistakes, patient complaints, and potential legal action especially during the COVID-19 pandemic.^43^ Burnout and work-related stress were two of the most recognised reasons for leaving among doctors.^63^

##### Failing organisational structure

In terms of organisational issues within the NHS, studies described limited opportunities for career development or promotion,^3,44^ lack of research funding opportunities,^42^ poor access to training,^43^ a perception that training in the UK was inadequate,^65^ and an inability to meet the requirements for revalidation because work-related time pressures would not allow for continuing professional development.^56^ One study noted that promotion appeared to be based more on the length of time an employee had served, rather than capability.^19^

The outlook of working for the NHS was described as bleak in one study^44^ with health workers considering leaving due to dissatisfaction with their role, working conditions or environment^48,63,66^ or uncertainty over contracts being proposed.^61,65^ The proposed introduction of a 7-day working week had made some GPs consider leaving the NHS.^49,59^ Some participants described being uncertain of the future of their professions due to limited NHS funding^42^ and health workers reported leaving the NHS either because of their contracts not being renewed or their roles being made redundant.^3^ NHS working conditions lacked flexibility^19,62^ and professionals described a lack of autonomy^46^ and control of their working lives.^45^ Frequent organisational changes contributed to this, causing significant dissatisfaction.^64^

Some participants described managers and supervisors as generally unsupportive^3,45,52,56,62,65,66^ with some health workers lacking mentors.^48^ Amongst those who had decided to leave, some perceived there to be no interest or efforts in retaining them.^44^ Several studies cited a bullying culture within the NHS, which increased intentions to leave.^3,45,48,50,53,56,57^ Bullying was experienced from patients, colleagues, managers, and the wider organisation^50,56^ but was found to significantly increase turnover intention when it was experienced from colleagues, an effect that was not moderated by an organisational response.^50^

Health workers also reported leaving the NHS for financial reasons^42,43^ or lack of paid roles,^3^ but pay as a push factor was more frequently cited in the context of not being commensurate with the level of responsibility, work demands and the overall economy.^19,44,65^ When compared to other push factors, pay was ranked lower in importance to staff shortages and workload.^25^

##### Personal and emotional factors

Declining physical or mental health led some health workers to consider leaving the NHS or retire early.^56,62,64^ GPs who attended work while sick more than once in a 12-month period had a higher chance of leaving than those who did not attend when sick.^51^ Working in the NHS had a significant emotional impact on staff, characterised by feeling disillusioned, anxious, exhausted, and burnt out, all of which were secondary to work-related pressures.^57,59^ ‘Surface acting’ at work, where the health worker displays emotions that they know they do not have and suppress their real feelings for the purpose of the patient, was linked to higher levels of depersonalisation and emotional exhaustion, which increased turnover intentions.^41^

Respondents who reported a poor work/life balance^19,44,60,65^ and lower job satisfaction^47,49,54,57,59^ were more likely to leave than those reporting a good balance and high job satisfaction respectively. In one study, job satisfaction had a negative association with turnover intentions; that is the intention to leave increased as job satisfaction decreased or vice versa.^54^ One study measured on-the-job embeddedness (fit and sacrifice with the organisation) and off-the-job embeddedness (links, fit and sacrifice with the community). On-the-job embeddedness was negatively associated with nurses’ turnover intentions, and the association between job satisfaction and intention to leave was stronger when off-the-job embeddedness was high versus low.^54^ Diagnostic uncertainty (a subjective perception of an inability to provide an explanation of the patient’s health problem) as well as low relatedness (the desire to experience connectedness and belonging with others) were also noted to reduce job satisfaction.^46,51^

Increasing age and length of service was found to reduce the impact of overall workload, working conditions, and work-life flexibility on intentions to leave.^59^ Personal development was more important for GPs with greater than 10 years of service and GP principals.^59^ Working conditions were more important to males GPs, while work-life flexibility and personal development were more important to female GPs.^59^ Some people left because they were unable to work in preferred locations.^45^

Some job roles were over-reliant on volunteers while paid roles were limited due to lack of funding.^3^ This, along with lack of promotion,^19^ a negative portrayal of their profession by patients and politicians,^57,64,65^ being exposed to a poor working environment, overworking and being underappreciated^48^ all contributed to NHS staff reporting having a low morale and feeling undervalued.

##### Wider factors

Due to Brexit, foreign health workers reported feeling rejected and no longer welcome in the UK.^39^ Brexit was also perceived by some as racist, and impacted on doctors’ decisions to leave or consider leaving.^39,40,42^ Some health workers left due to perceived institutionalised racism within the NHS.^45,56^ Other push factors identified included negative portrayal of GPs in the media, unfavourable political factors in the NHS,^57,64,65^ a general rise in the cost of accommodation,^43^ and poor transport links.^43^

COVID-19 was mentioned as a reason for leaving the NHS in three studies. Out of 5639 participants in one study, 2.4% listed COVID-19 as one of their top three reasons for leaving.^56^ Participants in a second study stated leaving due to the increased workload caused by the pandemic.^43^ Workplace stress strengthened the desire of some health workers to retire prior to their state pension age, especially those who had spent greater than 16 years in post.^62^

### Pull factors

There was less evidence of pull factors compared to push factors in the studies included in this review. The pull factors associated with leaving the NHS either to work abroad or retire early were categorised into: personal reasons, and favourable organisational structures.

#### Personal reasons

Studies suggest that health workers leave the NHS for quality-of-life reasons.^5^ Respondents left to experience a better lifestyle abroad^65^ or to travel or work abroad for study or research,^59^ out of a need or desire to relocate^56,63^ or for family reasons.^61,63,65^ Work/life balance was also described as being better abroad.^42,61^

Health workers considered retiring early (while still young), or to increase time with family, for leisure and other personal interests or due to the retirement of a partner.^47,64^ Some left to care for a family member.^56^ Some GPs were also able to trade-off a full pension for early retirement and an adequate income, which some considered as a viable option.^64^ Health workers reported leaving medicine altogether because they were attracted to alternative careers,^45^ and because they had been influenced by their peers who had left.^45^ The sense of feeling better valued and recognised overseas was also noted.^42,44,48^

#### Favourable organisational structures

Finding alternative employment was the most common reason for leaving the NHS in one study.^3^ Health workers who had left the NHS to work abroad reported that the working conditions and environment were generally better in New Zealand and Australia^5,48^ with better research facilities and more opportunities for career development.^5,42^ Pay was perceived as better overseas,^5,42^ and health workers reported having more control over how services were delivered.^42^ Besides the prospect of greater opportunities, doctors also identified having to do less administrative work abroad, and being able to work at an appropriate skill level.^44^ From a logistical perspective, the process of obtaining a visa to work in Australia and New Zealand was found to be relatively easy to navigate.^48^

## Discussion

This systematic review aimed to identify and synthesise the push and pull factors influencing NHS-employed staff leaving or intending to leave the NHS. The most frequently cited push factor was NHS staff feeling overworked and pressured due to the nature and intensity of their workloads. Workload pressures were felt to be exacerbated by staff shortages as well as inadequate organisational structures to address these pressures. Other push factors included feeling undervalued and underpaid in relation to growing responsibilities. Whilst existing research on this topic has typically focused on individual NHS staff professions such as doctors or nurses, we sought to include studies of all NHS staff professions and specialities, finding common push and pull factors across all groups included in this review.

Given the far-reaching effects of feeling overworked, it was perhaps not surprising to find it was the most commonly cited reason for leaving or intending to leave the NHS. Excessive workloads appear to have a domino effect on staff, they lead to adverse health outcomes and low morale, which then lead to more staff leaving, reduced resources, and increased workload for those who remain, producing a vicious cycle^67^. Managing workload can be more difficult when flexible working is not available, due, for example, to workforce deficits and limited funding.^19^ A healthy work/life balance can mitigate some of these push factors, and this was noted as important to staff in several studies but difficult to attain due to the poor working conditions in the NHS and the lack of funding required to provide flexible working hours, which were frequently perceived as being better abroad. In one study, burnout was reversed when staff left the NHS and entered medical training programmes abroad^44^ suggesting that deficits in the organisational structure of the NHS could precipitate these poorer health outcomes. Further research is needed to explore the mechanisms behind feeling overworked and intention to leave, and retention strategies should focus on the factors within the organisational structure of the NHS that predispose staff to excessive workload levels.

Staff reported feeling undervalued while working in the NHS due: to roles being made redundant and outsourced to other organisations; salaries being downgraded; services being downsized as a result of funding cuts; and staff being expected to work for more unpaid hours.^3^ Exit interviews are not routinely done^44^; opportunities are therefore missed to understand why staff have chosen to leave and gather insights on how best to support staff, increase job satisfaction, and reduce turnover.

The relationship between pay and staff dissatisfaction with working in the NHS was tied to staff perceptions of their value. Pay was frequently cited as inadequate in relation to staff qualifications, experience, hours worked and level of responsibility. Low pay was mentioned in most included studies, but was only cited as the most common reason for staff leaving in one study,^42^ having relatively small influence on turnover intentions in most others. This suggests that NHS staff are concerned about fair pay relative to their job demands and level of expertise rather than receiving higher pay more generally. In the UK nurses’ industrial action December 2022, one of the major factors driving the decision to strike was a perceived pay stagnation in the NHS despite an increase in professional development, responsibility and workload.^4^ This suggests that retention strategies are likely to have a limited effect if they are focused on increasing pay alone, without addressing job demands and issues relating to workload, which have a stronger effect on staff leaving, as found in an earlier systematic review exploring the relationship between labour force satisfaction, wages, and retention within the NHS.^34^

A common trend amongst staff turnover intentions was a lack of opportunities for career development within the NHS. Training in the UK was described as being largely replaced by service provision,^44^ with progression being based on length of service rather than skill acquisition.^19^ Opportunities for career progression is a particularly decisive factor among older and more experienced health workers, with a trend of older doctors from various professions intending to leave well before retirement age, potentially due to a lack of opportunities for career progression. This risks the NHS losing more of their experienced staff and more junior staff having to work in an environment where support from senior colleagues is limited, which in turn can result in younger staff wanting to leave due to increased work-related pressures and lack of support. This was recognised by NHS as an issue for staff retention in their 2020/21 *People Plan*. Alternative training routes such as apprenticeships are one of the main strategies towards growing the NHS workforce,^12^ but the effectiveness of this strategy will depend on the availability of experienced staff to support trainees.

Whilst some studies reported the pull of early retirement or warmer climates in other countries, what is of greater significance to UK retention policies are studies reporting staff moving abroad for professional reasons. This included perceived better opportunities for career progression, availability of better working conditions, flexible hours, a reduction in administrative workload, and being able to work at their skill level and in their preferred profession abroad. Compared to the NHS, remuneration was noted to be better abroad. When comparing the flow of medical professionals between developed countries, the UK has been identified as a net exporter of doctors.^22^ Staff in the UK are leaving a health care system they perceive to be poorly organised in favour of alternative employment perceived as having better working conditions. As such, further research exploring perceptions of the desirable “pull” factors of other countries is needed help inform retention strategies. While the migration of health professionals from lower- and-middle- income nations to high income countries is well documented, relatively less is known about the drivers of migration between high income countries.

### Strengths and limitations

Existing research on this topic has typically focused on specific NHS professions, namely doctors and nurses, limiting the evidence for policies aiming to improve staff retention. Our review sought to include studies reporting push and pull factors of any NHS profession and covered a range of professions including nurses, midwives, dentists, counsellors, healthcare assistants, radiographers, groups of unspecified NHS healthcare workers, and various types of doctors. Whilst not all NHS professions are represented in our review, we found that across 10 professions, reasons for NHS staff either leaving or intending to leave appear to be common. Most of the studies, however, were based in England meaning our findings may not reflect experiences of NHS staff in other UK nations which may require different approaches. Furthermore, some of the studies measured intentions to leave at a given point in time, which may not directly translate to staff actually leaving. As data was collected retrospectively in the studies where respondents had left the NHS, there is a possibility of recall bias, where participants may have left out relevant details. There was a lack of data on reasons why staff may leave to work in private practice, and the perceived benefits of working in the private sector when compared to the NHS.

## Conclusion

NHS staff face several challenges which significantly affect their job satisfaction, health, and morale, which lead to staff intending to and actually leaving the NHS. While staff may retire early or move abroad due to personal reasons, the majority of factors responsible for high turnover are related to issues relating to the organisational structure within the NHS. Further research comparing the UK NHS with health systems of other high-income countries such as Australia and New Zealand are needed to inform potential changes to the structure of the NHS that would reduce turnover and improve retention.

## Supplemental Material


Supplemental material - Understanding why health professionals are leaving the UK national health service (NHS) – A systematic review and narrative synthesis
Supplemental material for Understanding why health professionals are leaving the UK national health service (NHS) – A systematic review and narrative synthesis by Chukwunwuba Richard Onyejesi, Tiffeny James and Kalpa Kharicha in Journal of Health Services Research & Policy
