# Supplementary material for: Understanding why health professionals are leaving the UK national health service (NHS) – A systematic review and narrative synthesis
Source: J Health Serv Res Policy. 2025 Oct 8;31(2):125–39. doi: 10.1177/13558196251384845 (PMC12988014; doi:10.1177/13558196251384845)
Supplement: Supplemental material - Understanding why health professionals are leaving the UK national health service (NHS) – A systematic review and narrative synthesis [file sj-pdf-1-hsr-10.1177_13558196251384845.pdf]

Journal of Health Services Research & Policy

Understanding why health professionals are leaving the UK National Health Service (NHS) – a systematic review and narrative synthesis.

Onyejesi CR, James T, Kharicha K.

Online Supplement 1

Table S1 – PRISMA checklist

Section and Topic	Item #	Checklist item	Location where item is reported
**TITLE**			
Title	1	Identify the report as a systematic review.	Page 1
**ABSTRACT**			
Abstract	2	See the PRISMA 2020 for Abstracts checklist.	Page 1
**INTRODUCTION**			
Rationale	3	Describe the rationale for the review in the context of existing knowledge.	Pages 5-6
Objectives	4	Provide an explicit statement of the objective(s) or question(s) the review addresses.	Page 6
**METHODS**			
Eligibility criteria	5	Specify the inclusion and exclusion criteria for the review and how studies were grouped for the syntheses.	Page 7
Information sources	6	Specify all databases, registers, websites, organisations, reference lists and other sources searched or consulted to identify studies. Specify the date when each source was last searched or consulted.	Pages 7-8
Search strategy	7	Present the full search strategies for all databases, registers and websites, including any filters and limits used.	Appendix 2 (pages 34 – 36)
Selection process	8	Specify the methods used to decide whether a study met the inclusion criteria of the review, including how many reviewers screened each record and each report retrieved, whether they worked independently, and if applicable, details of automation tools used in the process.	Page 8
Data collection process	9	Specify the methods used to collect data from reports, including how many reviewers collected data from each report, whether they worked independently, any processes for obtaining or confirming data from study investigators, and if applicable, details of automation tools used in the process.	Pages 8-9
Data items	10a	List and define all outcomes for which data were sought. Specify whether all results that were compatible with each outcome domain in each study were sought (e.g. for all measures, time points, analyses), and if not, the methods used to decide which results to collect.	Pages 8-9
	10b	List and define all other variables for which data were sought (e.g. participant and intervention characteristics, funding sources). Describe any assumptions made about any missing or unclear information.	N/A
Study risk of bias assessment	11	Specify the methods used to assess risk of bias in the included studies, including details of the tool(s) used, how many reviewers assessed each study and whether they worked independently, and if applicable, details of automation tools used in the process.	Page 9
Effect measures	12	Specify for each outcome the effect measure(s) (e.g. risk ratio, mean difference) used in the synthesis or presentation of results.	N/A

Synthesis methods	13a	Describe the processes used to decide which studies were eligible for each synthesis (e.g. tabulating the study intervention characteristics and comparing against the planned groups for each synthesis (item #5)).	Page 8
	13b	Describe any methods required to prepare the data for presentation or synthesis, such as handling of missing summary statistics, or data conversions.	Page 9
	13c	Describe any methods used to tabulate or visually display results of individual studies and syntheses.	Pages 8 - 9
	13d	Describe any methods used to synthesize results and provide a rationale for the choice(s). If meta-analysis was performed, describe the model(s), method(s) to identify the presence and extent of statistical heterogeneity, and software package(s) used.	Page 9
	13e	Describe any methods used to explore possible causes of heterogeneity among study results (e.g. subgroup analysis, meta-regression).	N/A
	13f	Describe any sensitivity analyses conducted to assess robustness of the synthesized results.	N/A
Reporting bias assessment	14	Describe any methods used to assess risk of bias due to missing results in a synthesis (arising from reporting biases).	N/A
Certainty assessment	15	Describe any methods used to assess certainty (or confidence) in the body of evidence for an outcome.	N/A
**RESULTS**			
Study selection	16a	Describe the results of the search and selection process, from the number of records identified in the search to the number of studies included in the review, ideally using a flow diagram.	Pages 9 & 10
	16b	Cite studies that might appear to meet the inclusion criteria, but which were excluded, and explain why they were excluded.	Page 9
Study characteristics	17	Cite each included study and present its characteristics.	Table 1 (pages 12-17)
Risk of bias in studies	18	Present assessments of risk of bias for each included study.	Appendices 4, 5, 6, & 7 (pages 39, 40, 41, & 42)
Results of individual studies	19	For all outcomes, present, for each study: (a) summary statistics for each group (where appropriate) and (b) an effect estimate and its precision (e.g. confidence/credible interval), ideally using structured tables or plots.	N/A
Results of syntheses	20a	For each synthesis, briefly summarise the characteristics and risk of bias among contributing studies.	N/A
	20b	Present results of all statistical syntheses conducted. If meta-analysis was done, present for each the summary estimate and its precision (e.g. confidence/credible interval) and measures of statistical heterogeneity. If comparing groups, describe the direction of the effect.	N/A
	20c	Present results of all investigations of possible causes of heterogeneity among study results.	N/A
	20d	Present results of all sensitivity analyses conducted to assess the robustness of the synthesized results.	N/A
Reporting biases	21	Present assessments of risk of bias due to missing results (arising from reporting biases) for each synthesis assessed.	N/A
Certainty of evidence	22	Present assessments of certainty (or confidence) in the body of evidence for each outcome assessed.	N/A

**DISCUSSION**			
Discussion	23a	Provide a general interpretation of the results in the context of other evidence.	Pages 23 - 26
	23b	Discuss any limitations of the evidence included in the review.	Pages 26 - 27
	23c	Discuss any limitations of the review processes used.	Pages 26 - 27
	23d	Discuss implications of the results for practice, policy, and future research.	Pages 23 - 26
**OTHER INFORMATION**			
Registration and protocol	24a	Provide registration information for the review, including register name and registration number, or state that the review was not registered.	Page 7
	24b	Indicate where the review protocol can be accessed, or state that a protocol was not prepared.	Page 7
	24c	Describe and explain any amendments to information provided at registration or in the protocol.	N/A
Support	25	Describe sources of financial or non-financial support for the review, and the role of the funders or sponsors in the review.	Title page
Competing interests	26	Declare any competing interests of review authors.	Title page
Availability of data, code and other materials	27	Report which of the following are publicly available and where they can be found: template data collection forms; data extracted from included studies; data used for all analyses; analytic code; any other materials used in the review.	Title Page

Journal of Health Services Research & Policy
Understanding why health professionals are leaving the UK National Health Service (NHS) – a systematic review and narrative synthesis.
Onyejesi CR, James T, Kharicha K.

Online Supplement 1
Table S2 – Search strategies

PubMed

S/N	Keywords	Number of Studies included
**1**	Anaesthetist[tw] OR ''allied health professionals''[tw] OR ''Ambulance care assistant''[tw] OR Audiologist[tw] OR ''Biomedical scientists''[tw] OR Biochemist[tw] OR consultant*[tw] OR Cardiologist[tw] OR cytologist[tw] OR Counsellor[tw] OR Dietitian[tw] OR dentist*[tw] OR Endocrinologist[tw] OR ''General Practitioner*''[tw] OR GP[tw] OR Gynaecologist[tw] OR Gastroenterologist[tw] OR "healthcare worker"[tw] OR "Health Personnel"[Mesh] OR ''Healthcare scientist''[tw] OR ‘’Healthcare support worker''[tw] OR ''Healthcare assistant''[tw] OR ''Haematologist''[tw] OR ''Medical doctor*''[tw] OR Midwife*[tw] OR Midwive* OR Microbiologist[tw] OR nurs*[tw] OR "nhs staff"[tw] OR Nephrologist[tw] OR Neurologist[tw] OR Neurosurgeon[tw] OR ‘’Occupational therapist’’[tw] OR Ophthalmologist[tw] OR Orthoptist[tw] OR physician*[tw] OR physiotherapist*[tw] OR pathologist[tw] OR Pharmacist*[tw] OR Psychologist*[tw] OR Psychiatrist*[tw] OR ''Public health practitioner*''[tw] OR Paramedic[tw] OR Podiatrist[tw] OR Paediatrician[tw] OR Prosthetist[tw] OR Phlebotomist[tw] OR Psychotherapist[tw] OR Radiologist[tw] OR radiographer*[tw] OR Surgeon*[tw] OR ''Speech and language therapist''[tw] OR Urologist[tw]	2216872
**2**	"Personnel Turnover"[Mesh] OR Resign*[tw] OR relocat*[tw] OR moving[tw] OR leav*[tw] OR ''intent to leave''[tw] OR ''intend to leave''[tw] OR migrat*[tw] OR emigrat*[tw] OR overseas[tw] OR motivat* OR ''early retirement''[tw] OR retention OR retire*[tw]	1320738
**3**	"State Medicine"[Mesh] OR ‘’National Health Service’’[tw] OR NHS[tw]	96485
**4**	''United Kingdom’’[tw] OR England[tw] OR ‘’Northern Ireland’’[tw] OR Scotland[tw] OR Wales[tw] OR "Britain"[tw] OR "UK"[tw]	540278
**5**	#1 AND #2 AND #3 AND #4	1805
**6**	#1 AND #2 AND #3 AND #4 Filter: (from 2013 – 2023)	798

CINAHL

S/N	Keywords	Number of Studies included
**1**	"Health personnel OR Anaesthetist OR ''allied health professionals'' OR ''Ambulance care assistant'' OR Audiologist OR ''Biomedical scientists'' OR Biochemist OR consultant* OR Cardiologist OR cytologist OR Counsellor OR Dietitian OR dentist* OR Endocrinologist OR ''General Practitioner*'' OR GP OR Gynaecologist OR Gastroenterologist OR"healthcare worker" OR"Health Personnel" OR ''Healthcare scientist'' OR ''Healthcare support worker'' OR ''Healthcare assistant'' OR''Haematologist'' OR''Medical doctor*'' OR Midwife* OR Midwive* OR Microbiologist OR nurs* OR ''nhs staff'' OR Nephrologist OR Neurologist OR Neurosurgeon OR ''Occupational therapist'' OR Ophthalmologist OR Orthoptist OR physician* OR physiotherapist* OR pathologist OR Pharmacist* OR Psychologist* OR	**896,950**

	Psychiatrist* OR ''Public health practitioner*'' OR Paramedic OR Podiatrist OR Paediatrician OR Prosthetist OR Phlebotomist OR Psychotherapist OR Radiologist OR radiographer* OR Surgeon* OR ''Speech and language therapist'' OR Urologist" OR (MH"Health Personnel+") OR (MH "Allied Health Personnel+") OR (MH "Physicians, Family") OR (MH "Allied Health Professions+")	
**2**	"Resign* OR relocat* OR moving OR leav* OR ''intent to leave'' OR ''intend to leave'' OR migrat* OR emigrat* OR overseas OR motivat* OR ''early retirement'' OR retention OR retire* OR ''personnel turnover''" OR (MH "Personnel Turnover") OR (MH "Retirement") OR (MH "Personnel Retention") OR (MH "Employment Termination") OR (MH"Attitude of Health Personnel+") OR (MH "Personnel Shortage+") OR (MH "Personnel Management+") OR (MH "Intention")	**434,536**
**3**	(MH "National Health Programs") OR "''National Health Service'' OR ''NHS''"	**67,506**
**4**	(MH "Great Britain+") OR (MH "United Kingdom+") OR (MH "Northern Ireland") OR "''United Kingdom'' OR Britain OR UK OR England OR ''Northern Ireland'' OR Scotland OR Wales OR ''Great Britain''" OR (MH "Scotland") OR (MH "England") OR (MH"Wales")	**322,910**
**5**	S1 AND S2 AND S3 AND S4	**4646**
**6**	S1 AND S2 AND S3 AND S4 Limiters – Publication Date: 2013 01 01-2023 12 31	**1836**

<u>Embase</u>

**S/N**	**Keywords**	**Number of Studies included**
**1**	(Anaesthetist or ''allied health professional'' or ''ambulance care assistant'' or audiologist or ''biomedical scientists'' or biochemist or consultant$ or cardiologist or cytologist or counsellor or dietitian or dentist$ or endocrinologist or ''general practitioner$'' or GP or gynaecologist or gastroenterologist or ''healthcare worker'' or ''health personnel'' or ''healthcare scientist'' or ''healthcare support worker'' or ''healthcare assistant'' or ''haematologist'' or ''medical doctor'' or midwife$ or midwive$ or microbiologist or nurs$ or ''nhs staff'' or nephrologist or neurologist or neurosurgeon or ''occupational therapist'' or ophthalmologist or orthoptist or physician$ or physiotherapist$ or pathologist or pharmacist$ or psychologist$ or psychiatrist$ or ''public health practitioner$'' or paramedic or podiatrist or paediatrician or prosthetist or phlebotomist or psychotherapist or radiologist or radiographer$ or surgeon$ or ''speech therapist'' or urologist).mp. [mp=title, abstract, heading word, drug trade name, original title, device manufacturer, drug manufacturer, device trade name, keyword heading word, floating subheading word, candidate term word]	**2,989,719**
**2**	(leav$ or attrition or resign$ or moving or relocat$ or ''personnel turnover'' or migrat$ or emigrat$ or motivat$ or ''intend to leave'' or ''intent to leave'' or ''early retirement'' or retire$ or retention).mp. [mp=title, abstract, heading word, drug trade name, original title, device manufacturer, drug manufacturer, device trade name, keyword heading word, floating subheading word, candidate term word]	**1,720,137**
**3**	(NHS or ''National Health Service'').mp. [mp=title, abstract, heading word, drug trade name, original title, device manufacturer, drug manufacturer, device trade name, keyword heading word, floating subheading word, candidate term word]	**130,024**

**4**	(''United Kingdom'' or England or Wales or Scotland or ''Northern Ireland'' or Britain).mp. [mp=title, abstract, heading word, drug trade name, original title, device manufacturer, drug manufacturer, device trade name, keyword heading word, floating subheading word, candidate term word]	**751,028**
**5**	1 and 2 and 3 and 4	**1659**
**6**	5 and 2013:2023.(sa_year).	**838**

Web of Science

**S/N**	**Keywords**	**Number of Studies included**
**1**	ALL=(Anaesthetist OR ''allied health professionals'' OR ''Ambulance care assistant'' OR Audiologist OR ''Biomedical scientists'' OR Biochemist OR consultant* OR Cardiologist OR<br>cytologist OR Counsellor OR Dietitian OR dentist* OR Endocrinologist OR ''General Practitioner*'' OR GP OR Gynaecologist OR Gastroenterologist OR "healthcare worker" OR<br>"Health Personnel" OR ''Healthcare scientist'' OR ''Healthcare support worker'' OR ''Healthcare assistant'' OR ''Haematologist'' OR ''Medical doctor*'')	1041511
**2**	ALL=(Midwife* OR Midwive* OR Microbiologist OR nurs* OR "nhs staff" OR Nephrologist OR Neurologist OR Neurosurgeon OR ''Occupational therapist'' OR Ophthalmologist OR orthoptists OR physician* OR physiotherapist* OR pathologist OR Pharmacist* OR Psychologist* OR Psychiatrist* OR ''Public health practitioner*'' OR Paramedic OR Podiatrist OR Paediatrician OR Prosthetist OR Phlebotomist OR Psychotherapist OR Radiologist OR radiographer* OR Surgeon* OR ''Speech and language therapist'' OR Urologist)	2083158
**3**	ALL=(resign* OR relocat* OR moving OR leav* OR ''intent to leave'' OR ''intend to leave'' OR<br>migrat* OR emigrat* OR overseas OR motivat* OR ''early retirement'' OR retention OR retire)	3260078
**4**	ALL=(''National Health Service'' OR NHS)	832364
**5**	ALL=(''United Kingdom'' OR England OR ''Northern Ireland'' OR Scotland OR Wales OR ''Britain'' OR "UK")	7572063
**6**	#1 OR #2	2963355
**7**	#3 AND #4 AND #5 AND #6	5252
**8**	#3 AND #4 AND #5 AND #6 and 2013 or 2014 or 2015 or 2016 or 2017 or 2018 or 2019 or<br>2020 or 2021 or 2022 or 2023 (Publication Years)	4106

Journal of Health Services Research & Policy
Understanding why health professionals are leaving the UK National Health Service (NHS) – a systematic review and narrative synthesis.
Onyejesi CR, James T, Kharicha K.

Online Supplement 1
Table S3 – Quality of mixed methods studies

	Adequate rationale for mixed methods to address the research question	Different components effectively integrated to answer the research question	Outputs of integration components adequately interpreted	Divergencies and inconsistences between qualitative and quantitative results adequately addressed	Different components adhere to the quality criteria of each tradition of the methods involved
Prosser & Achour, 2023	Y	Y	Y	Y	Y
Spooner et al, 2016	Y	Y	Y	Y	Y
Ryan et al, 2019	Y	Y	Y	Y	Y
Mills et al, 2023	Y	Y	Y	Y	Y
Wilson et al, 2021	Y	Y	Y	Y	Y
Doran et al, 2016	Y	Y	Y	Y	Y
Lambert et al, 2018	Y	Y	Y	Y	Y
Dale et al, 2015	Y	Y	y	Y	Y

Key: Y = Yes, N = No, CT = Can't tell

Journal of Health Services Research & Policy
Understanding why health professionals are leaving the UK National Health Service (NHS) – a systematic review and narrative synthesis.
Onyejesi CR, James T, Kharicha K.

Online Supplement 1
Table S4 – Quality of qualitative studies

	Qualitative approach appropriate	Data collection methods adequate	Findings adequately derived from the data	Interpretation of results sufficiently substantiated by data	Coherence between data sources, collection, analysis and interpretation
Napier & Clinch, 2019	Y	Y	Y	Y	Y
Nightingale et al, 2021	Y	Y	Y	Y	Y
Milner et al, 2021a	Y	Y	Y	Y	Y
Pathmanathan & Snelling, 2023	Y	Y	Y	Y	Y
Smith et al, 2018	Y	Y	Y	Y	Y
Sansom et al., 2016	Y	Y	Y	Y	Y

Key: Y = Yes, N = No, CT = Can't tell

Online Supplement 1
Table S5 – Quality of quantitative descriptive studies

	Sampling strategy relevant to address the research question	Sample representative of the target population	Appropriate measurements	Low risk of non-response bias	Statistical analysis appropriate to answer question
Fasbender et al, 2019	Y	CT	Y	N	Y
Payne et al, 2023	Y	CT	Y	N	Y
Milner et al, 2021b	Y	N	Y	Y	Y
Crossland et al, 2021	Y	Y	Y	Y	Y
Weyman et al, 2023b	Y	CT	Y	Y	Y
Smith et al, 2017	Y	Y	Y	Y	Y
Owen et al, 2018	Y	Y	Y	Y	Y
Cheng et al, 2020	Y	Y	Y	Y	Y
Zhou et al, 2022	Y	Y	Y	Y	Y
Robson & Robson, 2016	Y	Y	Y	Y	Y
Nursing & Midwifery Council, 2020	Y	Y	Y	N	Y
Leversidge, 2016	Y	Y	Y	CT	Y
Gauld & Horsburgh, 2015	Y	Y	Y	N	Y
General Medical Council, 2021	Y	Y	Y	CT	Y

Key: Y = Yes, N = No, CT = Can’t tell

Journal of Health Services Research & Policy
Understanding why health professionals are leaving the UK National Health Service (NHS) – a systematic review and narrative synthesis.
Onyejesi CR, James T, Kharicha K.

Online Supplement 1
Table S6 – Quality of quantitative non-randomised studies

	Participants representative of target population	Appropriate measurements for both the outcome and intervention/exposure	Complete outcome data	Confounders accounted for in design and analysis	Exposure occurred as intended during study period
Theodosius et al, 2021	Y	Y	CT	CT	Y
Hood & Patton, 2022	Y	Y	Y	Y	Y
Khan et al, 2018	Y	Y	Y	Y	Y
Ocean & Meyer, 2023	Y	Y	Y	CT	Y

Key: Y = Yes, N = No, CT = Can't tell
